# Hemodynamic Effects of the Non-Peptidic Angiotensin-(1-7) Agonist AVE0991 in Liver Cirrhosis

**DOI:** 10.1371/journal.pone.0138732

**Published:** 2015-09-25

**Authors:** Sabine Klein, Chandana B. Herath, Robert Schierwagen, Josephine Grace, Tom Haltenhof, Frank E. Uschner, Christian P. Strassburg, Tilman Sauerbruch, Thomas Walther, Peter W. Angus, Jonel Trebicka

**Affiliations:** 1 Department of Internal Medicine I, University of Bonn, Bonn, Germany; 2 Department of Medicine, University of Melbourne, Austin Health, Heidelberg, Victoria, Australia; 3 Department of Obstetrics, Centre for Perinatal Medicine, Division of Women and Child Health, University of Leipzig, Leipzig, Germany; 4 Department of Pharmacology and Therapeutics, University College Cork, Cork, Ireland; 5 Department of Gastronenterology and Hepatology, Austin Health, Heidelberg, Victoria, Australia; University of Navarra School of Medicine and Center for Applied Medical Research (CIMA), SPAIN

## Abstract

**Background & Aims:**

Although in cirrhosis with portal hypertension levels of the vasoconstrictor angiotensin II are increased, this is accompanied by increased production of angiotensin (Ang)-(1–7), the endogenous ligand of the Mas receptor (MasR), which blunts hepatic fibrosis and decreases hepatic vascular resistance. Therefore, we investigated the effects of the non-peptidic Ang-(1–7) agonist, AVE0991, in experimental cirrhosis.

**Methods:**

Cirrhosis was induced by bile duct ligation (BDL) or carbon tetrachloride (CCl_4_) intoxication. The coloured microsphere technique assessed portal and systemic hemodynamic effects of AVE0991 *in vivo*. Hepatic expression of eNOS, p-eNOS, iNOS, JAK2, ROCK and p-Moesin were analyzed by western blots. Activities of ACE and ACE2 were investigated fluorometrically. Moreover, fibrosis was assessed in BDL rats receiving AVE0991.

**Results:**

*In vivo*, AVE0991 decreased portal pressure (PP) in both rat models of cirrhosis. Importantly, systemic effects were not observed. The hepatic effects of AVE0991 were based on upregulation of vasodilating pathways involving p-eNOS and iNOS, as well as by downregulation of the vasoconstrictive pathways (ROCK, p-Moesin). Short-term treatment with AVE0991 decreased the activity of ACE2, long-term treatment did not affect hepatic fibrosis in BDL rats.

**Conclusions:**

The non-peptidic agonist of Ang-(1–7), AVE0991, decreases portal pressure without influencing systemic pressure. Thus, although it does not inhibit fibrosis, AVE0991 may represent a promising new therapeutic strategy for lowering portal pressure.

## Introduction

The renin-angiotensin system (RAS) plays a pivotal role in cirrhosis and portal hypertension. Increased hepatic and systemic levels of angiotensin (Ang) II activates profibrotic and contractile pathways downstream of the AngII type 1 receptor (AT1R) on hepatic stellate cells (HSCs), promoting fibrosis and contraction. This, in turn, results in an increased vascular resistance [1–8].

**Fig 1:**
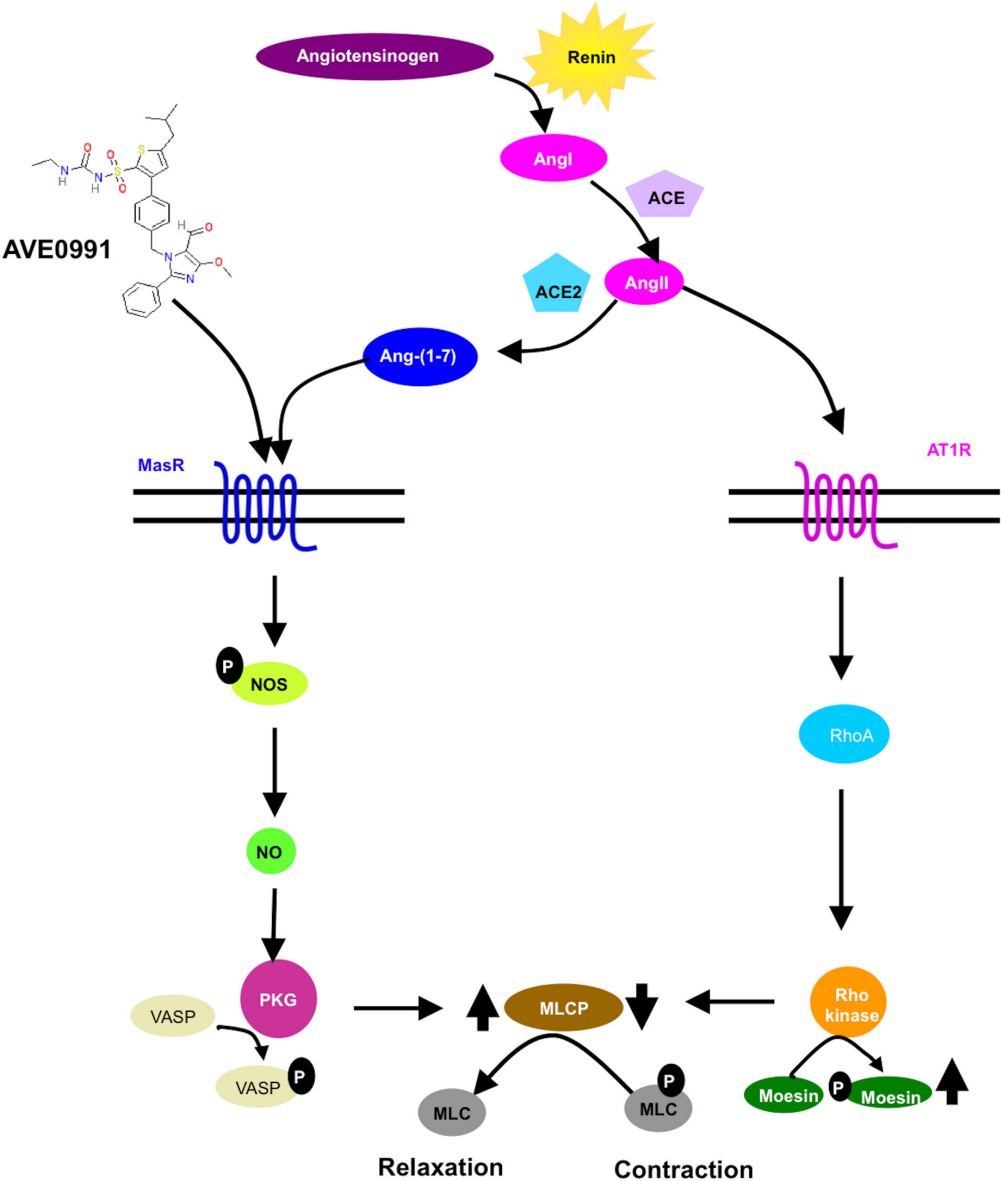
Schematic signalling pathways after formation of AngII and Ang-(1–7). In the RAS angiotensinogen is cleaved by renin to AngI. AngI is cleaved on the one side to AngII, a major vasoconstrictor and ligand of the AT1R. In the following JAK2 activates RhoA. Subsequently, the myosin light chain phosphatase (MLCP) is inhibited, which promotes cell contraction. On the other side, AngII can be cleaved by ACE2 to Ang-(1–7), the ligand of the MasR. The MasR mediates vasodilatation via NO and PKG, which activates the MLCP leading to relaxation. The marker for PKG is VASP-phosphorylation.

Ang-(1–7), a metabolite of AngII, counterbalances the effects of AngII in cirrhosis, blunting collagen production and causing vasodilatation in the splanchnic circulation (Fig 1) [9–11]. We recently showed that in cirrhotic animals with portal hypertension Ang-(1–7) production is increased in splanchnic vessels. This induces splanchnic vasodilatation by increasing nitric oxide (NO) production [1], and thus increases portal venous inflow to the liver. In addition, Ang-(1–7) also elicits intrahepatic vasodilatation [1,12]. Ang-(1–7) acts on the G protein-coupled receptor Mas (MasR), but might also interact with the Ang II type 2 receptor (AT2R) and a novel receptor subpopulation or combined receptors, since the vasodilatory effects are not solely blocked by the MasR antagonist A779 but also by D-Pro^7^-Ang-(1–7) [4,12,13]. From a clinical perspective, however, the utility of Ang-(1–7) is limited, since it cannot be given orally and due to its short biological half-life in circulation [14].

AVE0991 is a non-peptidic specific Ang-(1–7) agonist which can be given orally and has a much more prolonged half-life than the heptapeptide. In the kidney and lung, AVE0991 induces the release of nitric oxide (NO) and acts as an anti-inflammatory and antifibrotic agent via MasR stimulation [15–19]. Studies reporting the use of AVE0991 in liver disease are scarce, we have previously shown *in vitro* that AVE0991 reduced hepatic stellate cell (HSC) activation and collagen production [9].

Thus, we evaluated a potential therapeutic efficacy of long-acting MasR stimulation in cirrhosis models *in vivo* with regard to portal hypertension in two different models of cirrhosis in rats.

## Material and Methods

### Animals and models of liver disease

#### Animals

We used 89 Sprague-Dawley wild type (WT) rats. Experimental procedures were approved by the Animal Ethics Committee of Austin Health and of North Rhine-Westphalia (LANUV 84–02.04.2011.A164). WT rats were housed in a controlled environment (12 hour light/dark, temperature 22°C to 24°C) and fed standard rat chow *ad libitum* (Norco, Lismore NSW, Australia; Ssniff, Soest, Germany) with free access to water.

#### Toxic model

Eight rats (100g) underwent twice weekly inhalation of 1l/min CCl_4_ for 14–16 weeks until ascites was present as described previously [20,21]. Ten age-matched control rats did not receive CCl_4_.

#### Cholestatic model

BDL was performed in 40 rats (180g) after anesthesia with ketamine/xylazine (78mg/kg and 10mg/kg body weight) as previously described [12]. BDL rats were compared with 21 sham-operated rats.

### Hemodynamic studies

#### In-vivo hemodynamic studies

When rats developed ascites as a definite sign for the presence of portal hypertension, rats were used for the hemodynamic studies as described previously [1]. To assess the acute effect of AVE0991 invasive measurements of mean arterial pressure (MAP) and portal pressure (PP) were performed in cirrhotic rats. AVE0991 was administered at a dose of 1mg/kg via the femoral vein.

#### Microsphere technique

Briefly rats were anesthetized with ketamine/xylazine (78mg/kg and 10mg/kg body weight), respectively. To investigate hemodynamics, the coloured microsphere technique was performed as described previously [1]. Before and 1h after injection of AVE0991, 300.000 systemic (red/white) microspheres (15μm diameter, Triton-Technologies, San Diego, USA) were injected in the left ventricle. Mesenteric portal-systemic shunt volume was estimated before and after injection of 150.000 microspheres (yellow/blue) in the ileocecal vein [1]. Animals were sacrificed by a lethal dose of ketamine under anesthesia.

### Expression and activation levels

#### Western blot

Cirrhotic rat livers were collected from untreated sham and BDL operated rats and from CCl_4_ intoxicated WT rats. Additionally we collected pieces of AVE0991 treated rat livers directly after performing the microsphere technique. Western blot analysis (for antibodies see Table B in S1 Tables) was performed as described previously [20–23].

#### RT-PCR analysis

Immediately after the completion of experiments, wet liver weight was measured and a sample was snap-frozen in liquid nitrogen and stored at -80°C until extracted for RNA. Total RNA was extracted using TRI reagent (Sigma-Aldrich, Sydney, Australia) and reverse transcribed to cDNA by use of a protocol previously described [24] and in S1 Methods (for assays see Table A in S1 Tables).

#### ACE/ACE2 activity assay

Referring to Maul et al. [25] the activity of angiotensin converting enzyme (ACE) was measured using the fluorimetric method with the substrate Hip-His-Leu and the standard His-Leu. ACE2 activity was measured using the substrate Mca-APK (Dpn) and the fluorogenic control peptide as standard (Omni MMP^TM^) [25,26]. The activity of ACE was expressed as nmol His-Leu/min/mg protein and ACE2 activity as pmolMca-APK/min/mg protein.

### Fibrosis study

#### Administration of AVE0991

Osmotic mini-pumps (Model 2002, Alzet, Cupertino, CA, USA) were filled with AVE0991 (a gift from Sanofi-Aventis Deutschland GmbH, Germany) dissolved in polyethylene glycol 400 (Sigma-Aldrich, Sydney, Australia) and sterile water. The pumps were filled with 200μl of AVE0991 solution and incubated in saline overnight at 37°C were implanted in 14 rats in the peritoneal cavity during BDL surgery and delivered 28μg/kg/h for 2 weeks as previously described [2]. Twelve BDL rats were implanted with pumps containing vehicle alone and served as BDL control group. Sixteen sham-operated rats were also included. Animals were sacrified and livers analyzed 2 weeks after BDL and AVE0991 administration.

#### Hepatic hydroxyproline content

In analog segments (200mg) of snap-frozen livers, the hepatic hydroxyproline content was determined colorimetrically as described previously [27] and in S1 Methods.

### Histological analysis

#### Sirius-red staining

For the detection of collagen fibers, paraffin-embedded sections of liver (4μm) were stained with picrosirius-red and the proportion of Sirius-red staining per field at was assessed at ×100 magnification in a total of ten fields per section. This was performed in a blinded fashion using computerized image capture (MCID; Imaging Research, Cambridge, UK), as described previously [28].

#### Ductal proliferation

Bile ductular proliferation was scored using the grading system [29] detailed in S1 Methods.

### Statistical analysis

Data are presented as mean±standard error of the mean (s.e.m.). Student’s t-test was used for comparison where appropriate; Mann-Whitney-U test was used for comparison between groups. *p*-values < 0.05 were considered statistically significant.

## Results

### Portal and systemic effects of AVE0991 in cirrhosis

**Fig 2:**
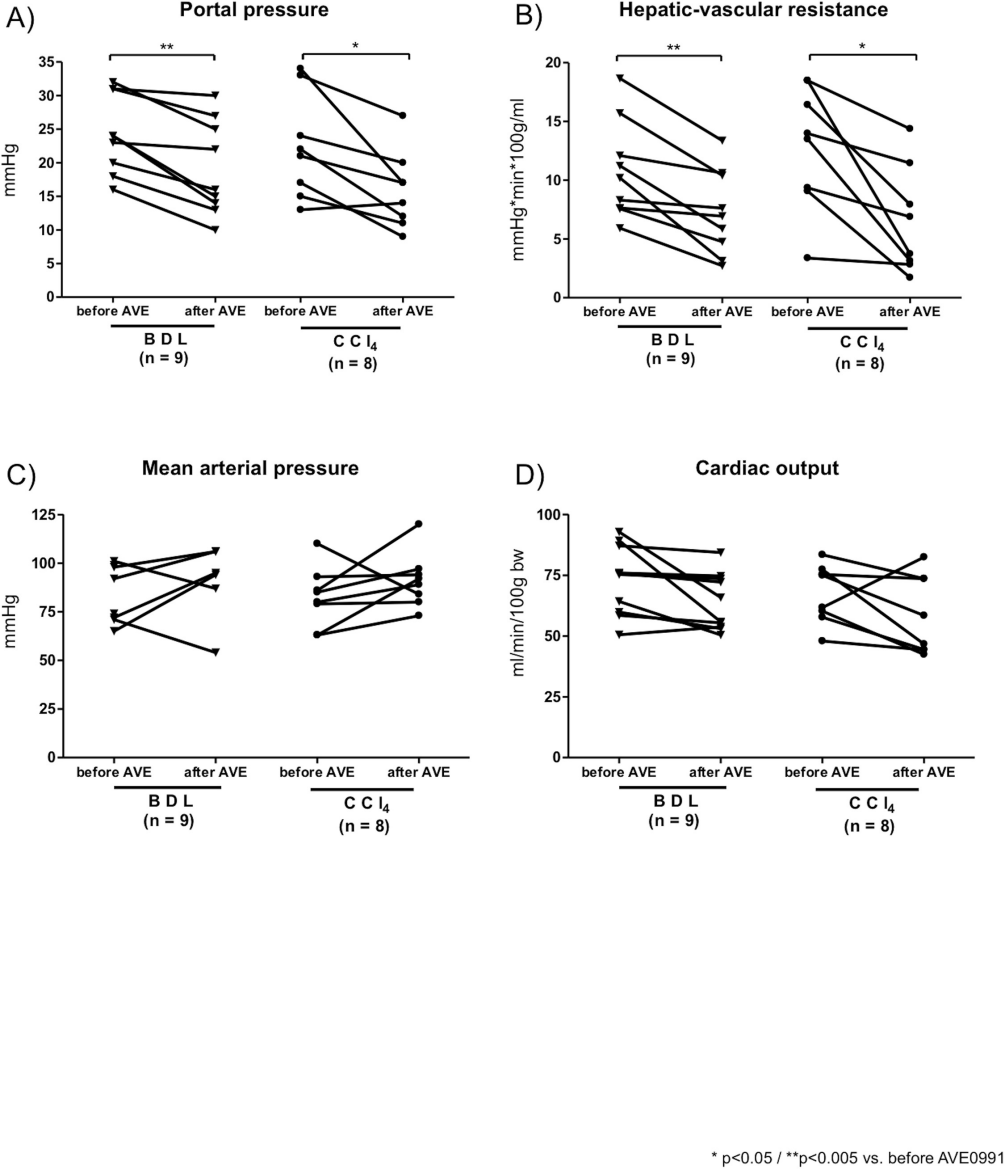
Acute portal and systemic effects of AVE0991 in cirrhosis. (A) Portal pressure was significantly decreased in cirrhotic BDL and CCl_4_ intoxicated rats by the acute i.v. injection of AVE0991 (1mg/kg). (B) The hepatic-vascular resistance dropped after the acute injection of 1mg/kg AVE0991 in all cirrhotic rats. (C) Acute AVE0991 injection did not influence the mean arterial pressure in BDL nor in CCl_4_ intoxicated rats. (D) The cardiac output was not changed after the acute i.v. injection of AVE0991 in all cirrhotic rats.

For *in vivo* hemodynamics we used the BDL (4–5 weeks) and the CCl_4_ intoxication (14–16 weeks) models when the presence of ascites indicated the presence of severe portal hypertension. Cirrhotic rats (BDL, CCl_4_) showed the typical signs of cirrhosis and portal hypertension with an increased portal pressure compared to controls (Table C in S1 Tables). Portal pressure was significantly decreased one hour after i.v.-injection of AVE0991 in cirrhotic rats (Fig 2A). The decrease in portal pressure after AVE0991 injection was associated with a significant drop in hepatic-vascular resistance in both rat models of cirrhosis (Fig 2B; Table C in S1 Tables), as well as with a increase in splanchnic vascular resistance (Table C in S1 Tables). AVE0991 did not change portal pressure and hepatic vascular resistance in control rats (Table C in S1 Tables).

AVE0991 injection did not affect the mean arterial pressure and cardiac output in control and cirrhotic rats (Fig 2C and 2D; Table C in S1 Tables). Therefore, stimulation of the MasR with AVE0991 did not induce systemic effect in cirrhosis.

### Hepatic expression of vasodilatatory and vasoconstrictory proteins in cirrhosis

**Fig 3:**
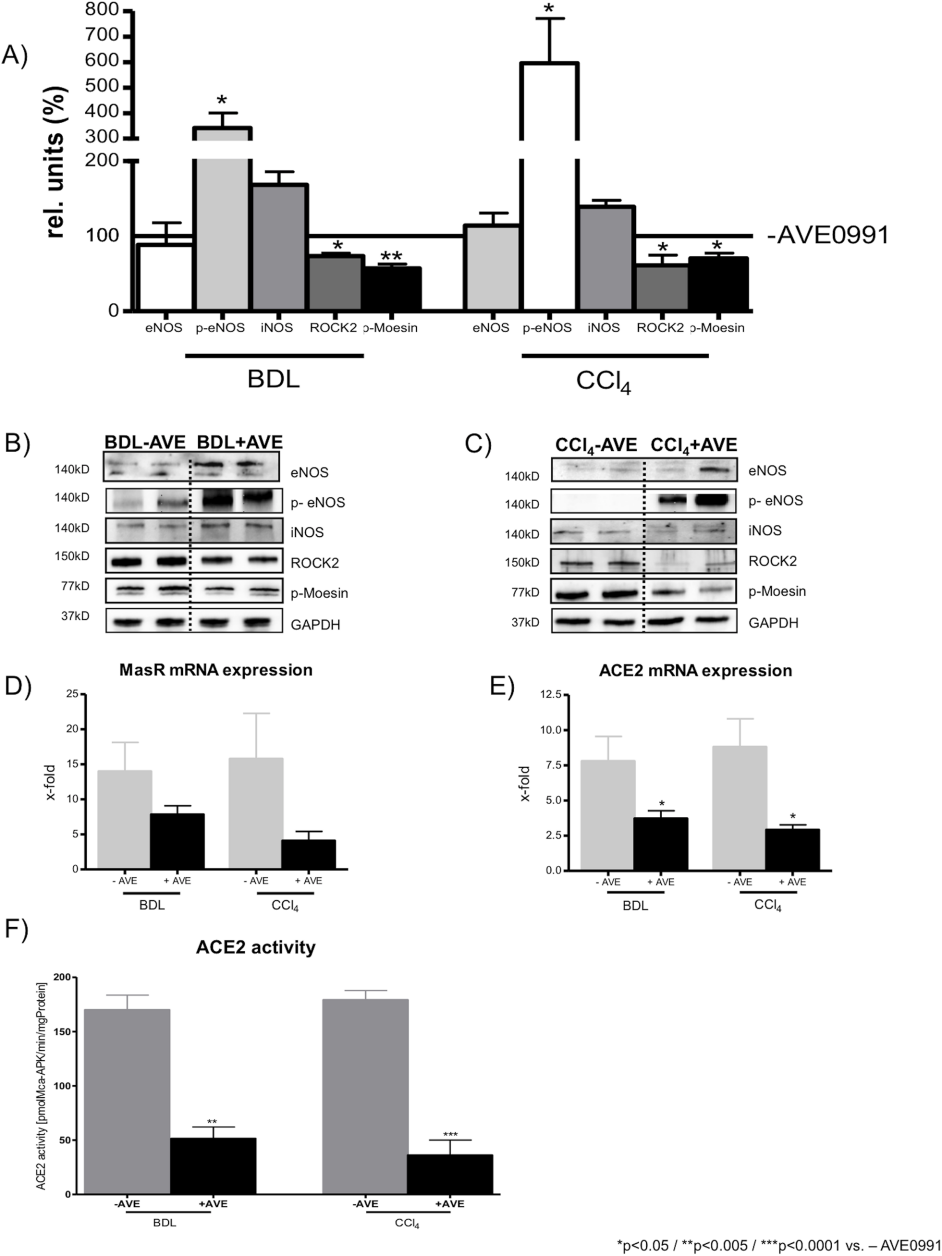
Hepatic expression of vasodilatatory and vasoconstrictory proteins, gene expression levels of the MasR and ACE2 and the hepatic ACE2 activity in cirrhosis with and without acute AVE0991 treatment. **(**A) Hepatic protein expression of the endothelial NO synthase (eNOS) was not influenced by AVE0991 injection in BDL rats. Its active form p-eNOS was significantly increased in BDL and CCl_4_ intoxicated rats after AVE0991 injection. The hepatic inducible NOS (iNOS) was not changed significantly by AVE0991 injection in cirrhotic rats. The vasoconstrictory Rho-kinase (ROCK) protein and its activity, measured by the phosphorylation of its substrate Moesin (pMoesin), were decreased significantly after AVE0991 injection in BDL and CCl_4_ intoxicated rats. (B, C) Representive blots of protein expression levels (eNOS, p-eNOS, iNOS, ROCK, p-Moesin, GAPDH) in cirrhotic BDL and CCl_4_ intoxicated rats with and without AVE0991 injection. (D) The hepatic mRNA expression of the Mas receptor was slightly decreased in BDL and CCl_4_ intoxicated rats after AVE0991 injection but not significantly. (E) The mRNA expression of ACE2 was significantly decreased in livers of BDL and CCl_4_ intoxicated rats after AVE0991 injection. (F) The injection of AVE0991 induced a significant reduction of the hepatic ACE2 activity in all cirrhotic rats. All results of mRNA expression levels are shown as the quantification of qRT-PCR results, normalized to sham without AVE0991.

Following AVE0991 injection, cirrhotic BDL and CCl_4_ intoxicated rats with portal hypertension, showed no significant change in protein expression levels of hepatic eNOS, but increased hepatic protein levels of phosphorylated eNOS (p-eNOS) underlining the mechanism for the hepatic vasodilation in cirrhosis (Fig 3A–3C). By contrast, hepatic iNOS expression levels were not significantly influenced one hour after acute AVE0991 injection in cirrhotic rats. Sham-operated rats showed also no change in hepatic eNOS expression level of after AVE0991 injection, but hepatic iNOS expression levels were significantly decreased in sham-operated rats after AVE0991 injection (Fig A in S1 Figs).

Hepatic expression levels of the vasoconstrictory proteins Rho-kinase (ROCK) and its activity, measured by the phosphorylation of its substrate Moesin, were significantly reduced after acute injection of AVE0991 in both models of cirrhotic rats, as well as in sham-operated control rats (Fig 3A–3C; Fig A in S1 Figs).

One hour after AVE0991 injection, the hepatic ACE2 mRNA expression was significantly reduced in rats of both cirrhosis models (Fig 3E), while hepatic MasR mRNA levels showed only a trend towards lower transcription after AVE (Fig 3D). Moreover, the reduction of ACE2 mRNA levels after AVE0991 injection was subsequently reflected by decreased ACE2 activity in BDL and CCl_4_ intoxicated rats (Fig 3F).

### Expression of vasodilatatory and vasoconstrictory proteins in aortas and hearts of cirrhotic rats

**Fig 4:**
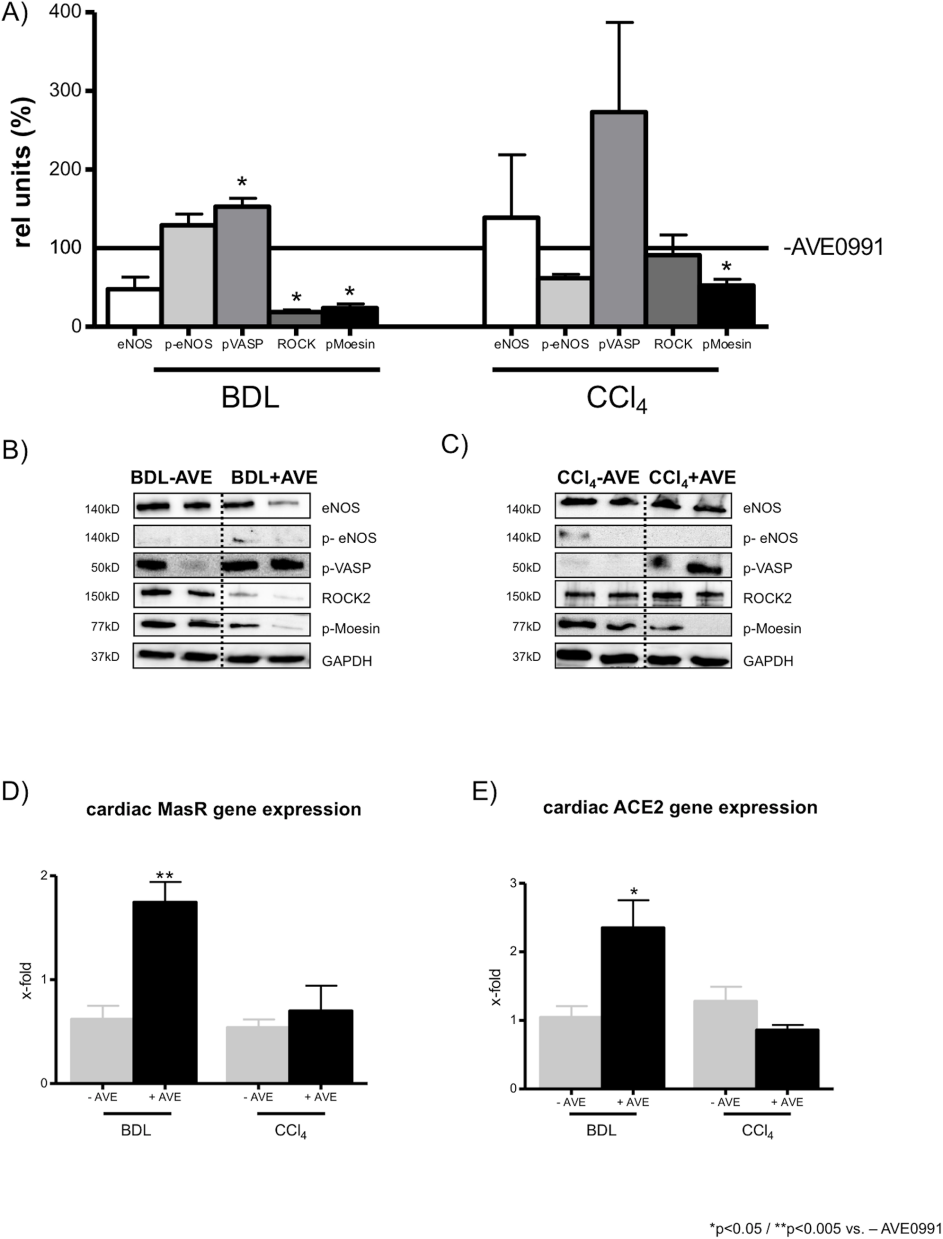
Cardiac expression levels of vasodilatatory and vasoconstrictory proteins and cardiac gene expression levels of the MasR and ACE2 in cirrhotic rats. (A) In hearts of BDL and CCl_4_ intoxicated rats the AVE0991 injection did not change the protein expression of eNOS significantly in BDL and CCl_4_ intoxicated rats. The protein expression of p-eNOS was not influenced by AVE0991 injection in all cirrhotic rats. The injection of AVE0991 increased the protein expression of pVASP in cirrhotic BDL rats, but not in CCl_4_ intoxicated rats. The expression level of ROCK and its activity, measured by the phosphorylation of its substrate Moesin, were significantly decreased by AVE0991 in BDL rats. In CCl_4_ intoxicated rats only the protein expression of pMoesin was reduced significantly after AVE0991 injection. (B, C) Representive blots of eNOS, p-eNOS, p-VASP, ROCK, p-Moesin and GAPDH in hearts of BDL and CCl_4_ intoxicated rats with and without AVE0991 injection. (D) The mRNA expression of the MasR was significantly increased in hearts of BDL rats after AVE0991 injection, but not in hearts of CCl_4_ intoxicated rats. (E) The cardiac mRNA expression of ACE2 was increased after AVE0991 injection in BDL rats but not changed in CCl_4_ intoxicated rats.

AVE0991 did not influence the cardiac expression levels of the vasodilatatory proteins eNOS and p-eNOS in cirrhotic or sham-operated control rats (Fig 4A and 4B; and Fig A in S1 Figs). However, phosphorylated VASP protein was increased significantly after AVE0991 injection in cirrhotic BDL rats, but not in CCl_4_ intoxicated rats (Fig 4A–4C).

Similarly to hepatic protein expression levels, cardiac ROCK2 and pMoesin expression levels were decreased after AVE0991 injection in cirrhotic BDL and CCl_4_ intoxicated rats, but not in sham-operated rats (Fig 4A–4C; and Fig A in S1 Figs).

AVE0991 induced significantly more cardiac MasR mRNA expression in BDL rats, but not in CCl_4_ intoxicated rats (Fig 4D). Similarly, cardiac ACE2 mRNA levels were increased in BDL, but not in CCl_4_ intoxicated rats after AVE0991 administration (Fig 4E). The protein levels of cardiac ACE were not changed by acute AVE0991 injection in sham operated or in CCl_4_ intoxicated rats. However, in cirrhotic BDL rats, AVE0991 increased significantly the cardiac ACE mRNA expression (Fig B in S1 Figs).

### Chronic effects of AVE0991 on BDL induced liver fibrosis

**Fig 5:**
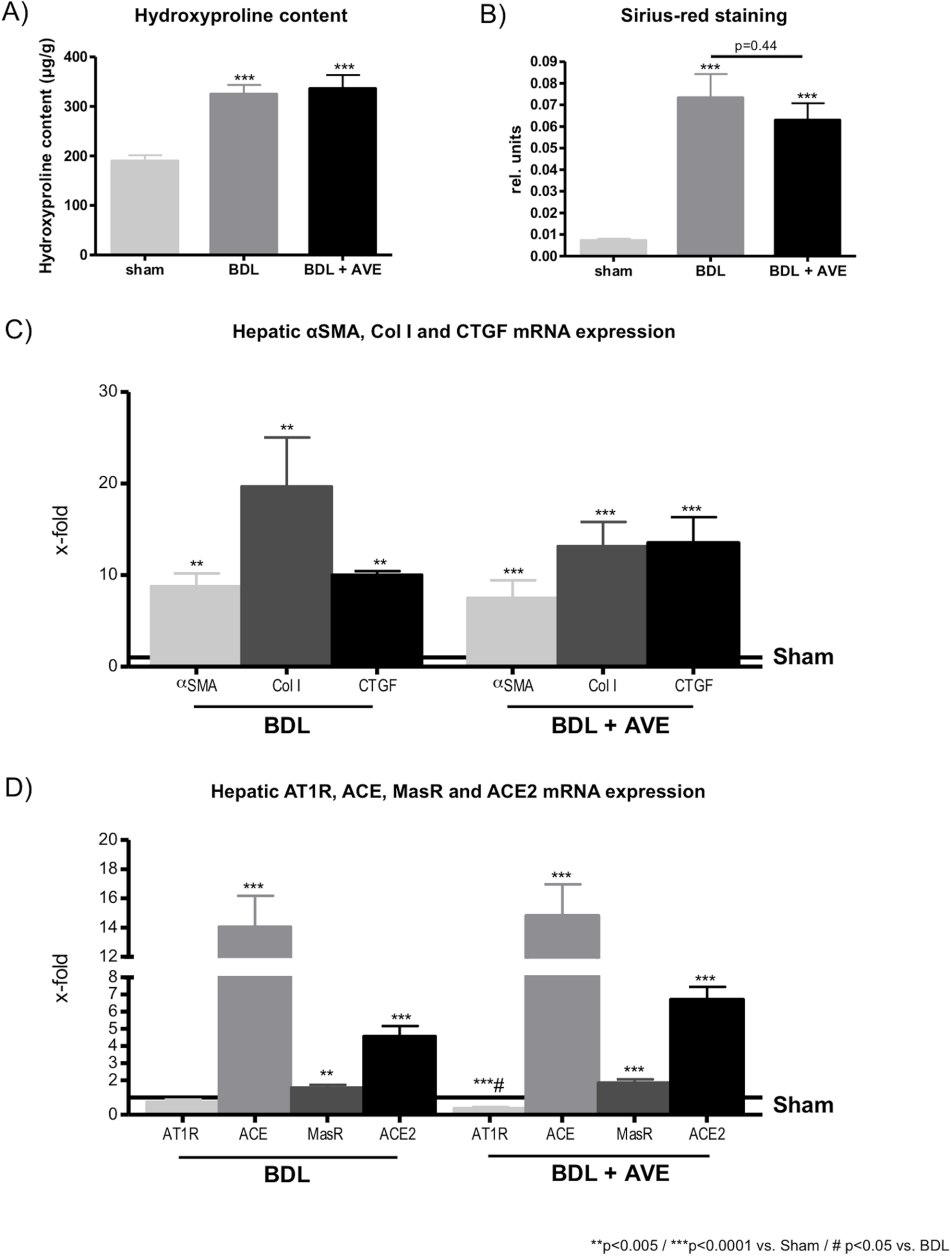
Chronic effect of AVE0991 on liver fibrosis and hepatic gene expression in liver cirrhosis. (A) The hydroxyproline content was increased in fibrotic rats after 2 weeks of BDL. The concomitant administration of AVE0991 by implanted mini-pumps (i.p.) did not change the hydroxyproline content compared to BDL rats without AVE0991. (B) Two weeks after BDL, the hepatic sirius-red staining was increased significantly. The concomitant administration of AVE0991 by osmotic mini-pumps did not influence the hepatic sirius-red staining compared to fibrotic BDL rats. (C) The hepatic genes αSMA, Col I and CTGF were increased in fibrotic rats, two weeks after BDL operation. The chronic administration of AVE0991 did not lower the mRNA expression levels of αSMA, Col I and CTGF compared to BDL livers without AVE0991 administration. (D) The hepatic mRNA level of AT1R was not changed in fibrotic BDL rats compared to sham-operated controls. After the administration of AVE0991 via osmotic mini-pumps, the AT1R mRNA expression was significantly reduced in fibrotic BDL rats compared to sham-operated and to BDL rats without AVE0991 administration. ACE mRNA expression levels were increased in untreated and AVE0991 treated BDL rats compared to sham-operated controls. Livers of BDL rats without and with AVE0991 administration express more MasR mRNA compared to sham-operated rats. The hepatic ACE2 mRNA expression levels were increased in BDL rats and in BDL rats after chronic AVE0991 administration compared to controls.

Two weeks after BDL hepatic hydroxyproline content increased significantly, and chronic treatment with AVE0991 via osmotic min-pumps did not change hepatic hydroxyproline content in fibrotic BDL rats (Fig 5A). As expected, Sirius-red staining was also increased in fibrotic BDL rat livers compared to sham operated rats. But again, the infusion of AVE0991 for two weeks did not reduce the level of Sirius-red staining (Fig 5B). Expression analyses of fibrotic BDL livers showed significantly increased hepatic αSMA, Col I, and CTGF mRNA levels compared to sham-operated control rats (Fig 5C). The chronic administration of AVE0991 did not change the mRNA expression levels of these markers (Fig 5C). Livers of fibrotic BDL rats with portal hypertension expressed significantly more mRNA of ACE, MasR and ACE2 (Fig 5D). After AVE0991 administration, AT1R mRNA expression was significantly reduced compared to sham-operated and to BDL rats without chronic AVE0991 administration. In BDL rats, hepatic expression levels of ACE, MasR and ACE2 mRNA were not affected by AVE0991 administration for 2 weeks compared to BDL rats with no AVE0991 treatment (Fig 5D).

Fibrotic BDL rats (2 weeks) showed higher bile ductular proliferation score compared to sham-operated rats, however administration of AVE0991 via osmotic mini-pumps for 2 weeks had no effect on the biliary proliferation score in fibrotic BDL rats (Fig B in S1 Figs).

## Discussion

This study showed for the first time, that acute administration of the non-peptidic Ang-(1–7) agonist AVE0991 reduced portal pressure in cirrhotic rats without causing systemic side effects. This effect was associated with an up-regulation of the p-eNOS/NO pathway and an attenuation of the Rho-kinase pathway within the liver. The effects of AVE0991 are well characterized *in vitro* and *in vivo* in renal, cardiovascular and endothelial research [10,11,15,18,30–33], but the current study is the first to characterize its effects in cirrhosis *in vivo*.

We have recently reported that Ang-(1–7) lowers portal pressure in cirrhotic rats *in vivo* and liver perfusion pressure *ex vivo* in cirrhotic livers [1,12]. This was mediated by endothelial NOS [1,9]. It has been shown previously, that binding of AVE0991 to the MasR releases more NO then Ang-(1–7) [11]. Indeed, we constrained the significant increment of p-eNOS after AVE0991 treatment [11]. Therefore, the excessive NO production after stimulation of the MasR by AVE0991 might explain the strong portal pressure reducing effects in both cirrhosis models. These effects were paralleled by inhibition of the Rho-kinase pathway, which is linked to vasoconstrictive mechanisms downstream of the AT1R [2]. These molecular findings explains our observation that there was a significant reduction in intrahepatic-vascular resistance in two different cirrhotic rat models *in vivo*, leading to a significant reduction of portal pressure. Either a strong association of AT1R and MasR or crosstalks of their downstream pathways might explain these data. Further studies are required to highlight this interaction in detail.

Besides the upregulation of MasR in the cirrhotic liver, increased MasR expression was described in the splanchnic-vascular bed of cirrhotic animals and humans [1]. Indeed, recently we showed that Ang-(1–7) administration worsened mesenteric vasodilatation in cirrhotic rats, which might limit its clinical use although it reduces portal pressure. Contrary to the effects of Ang-(1–7) [1], the splanchnic-vascular resistance was not decreased by AVE0991 in cirrhotic rats. The vasodilatatory effect of the MasR agonist AVE0991 may be more prominent in the diseased liver and less in splanchnic-vascular tissues [1,34]. A putative explanation for the prominent hepatic effect of AVE0991 might be its elimination by the liver, as a nonpeptidic imidazol derivate; therefore it may have major hepatic effects before further glucuronidation [35]. By contrast, AVE0991 slightly increased splanchnic vascular resistance in our cirrhotic animals *in vivo*. This increase in splanchnic vascular resistance might be a direct AVE0991 effect (possibly via interaction with AT1R), or it might be a splanchnic vascular response to the marked decrease of intrahepatic resistance.

Furthermore, the systemic and peripheral circulation was not affected by AVE0991. This was surprising since AVE0991 has been described to be a potent vasodilator in different pathological extrahepatic conditions (e.g. atherosclerosis, cardiac, renal and pulmonary diseases) [11,19,30–33]. Though at the molecular level, we could observe some effects lowering the cardiac contractile potential in the model of cirrhotic cardiomyopathy, this was not mirrored by our functional hemodynamic measurements.

These differences between the effects of Ang-(1–7) and AVE0991 might be explained by a number of factors. In comparison to Ang-(1–7), the imidazol derivate AVE0991 has a longer half-life due to its resistance to proteolytic enzymes. The differences between AVE0991 and Ang-(1–7) are also illustrated by the fact that AVE0991 does not directly antagonize vasoconstrictive AngII effects in the liver [12], while the vasoconstrictive AngII effect is counteracted by Ang-(1–7) injection. This is likely due to the effects of Ang-(1–7), which are partly independent of MasR stimulation and thus, the MasR may not be the sole receptor mediating Ang-(1–7) action in the cirrhotic liver. Probably the receptor binding profile is different with AVE0991 only stimulating the Mas receptor and Ang-(1–7) stimulating other receptors such as AT2R and MrgD-receptor. Furthermore, we found that the hepatic ACE2 activity was decreased shortly after AVE0991 injection, which might lead to the hypothesis that MasR-stimulation blunts Ang-(1–7) formation by restricting ACE2 activity, and therefore the systemic production of Ang(1–7) with the subsequent systemic vasodilation.

In contrast to our previous *in vitro* work using HSCs that showed a blocking effect of AVE0991 on αSMA and collagen transcription [9], the current study suggests such that AVE0991 has no measurable antifibrotic activity *in vivo*. One possible speculative explanation might be, that AVE0991 blunts the expression and activity of ACE/MasR axis as shown in the acute experiments. However, in other models, AVE0991 might have an effect on hepatic fibrosis.

In summary, we could show that AVE0991 is a potential therapeutic agent to lower portal pressure without extrahepatic systemic effects. This nonpeptidic compound should be further investigated in patients with portal hypertension.

## Supporting Information

S1 Figs Supporting Figures A and B. (DOCX)

S1 Methods Supporting Material and Methods (DOCX)

S1 Tables Supporting Tables A, B and C. (DOCX)

## Abbreviations

αSMA: alpha smooth muscle actin
Ang-(1–7): Angiotensin-(1–7)
AngI: Angiotensin I
AngII: Angiotensin II
AT1R: Angiotensin II type 1 receptor
AT2R: Angiotensin II type 2 receptor
ACE: Angiotensin converting enzyme
ACE2: Angiotensin converting enzyme 2
BDL: bile duct ligation
CCl_4_: carbon tetrachloride
CTGF: connective tissue growth factor
Col I: collagen type 1
eNOS: endothelial nitric oxide synthase
HSC: hepatic stellate cells
iNOS: inducible nitric oxide synthase
JAK2: janus kinase 2
MasR: Mas receptor
MAP: mean arterial pressure
mRNA: messenger ribonucleic acid
MTX: methoxamine
MLCP: myosin light chain phosphatase
NO: nitric oxide
PKG: protein kinase G
p-eNOS: phosphorylated endothelial nitric oxide synthase
PP: portal pressure
RAS: renin-angiotensin system
ROCK: Rho-associated protein kinase
s.e.m.: standard error of the mean
