# Supplementary material for: Hemodynamic Effects of the Non-Peptidic Angiotensin-(1-7) Agonist AVE0991 in Liver Cirrhosis
Source: PLoS One. 2015 Sep 25;10(9):e0138732. doi: 10.1371/journal.pone.0138732 (PMC4583473; doi:10.1371/journal.pone.0138732)
Supplement: S1 Figs — (DOCX) [file pone.0138732.s001.docx]

Hemodynamic effects of the non-peptidic angiotensin-(1-7) agonist AVE0991 in liver cirrhosis

Sabine Klein¹*, Chandana B. Herath^2*^, Robert Schierwagen^1^, Josephine Grace^2^, Tom Haltenhof^3^, Frank E. Uschner^1^, Christian P. Strassburg¹, Tilman Sauerbruch^1^, Thomas Walther^3,4^, Peter W. Angus^5$^ and Jonel Trebicka¹^$+^

^*^ shared first authorship; ^$^ shared last authorship; ^+^ Corresponding author

**Affiliations:**

¹Department of Internal Medicine I, University of Bonn, Germany

^2^Department of Medicine, University of Melbourne, Austin Health, Heidelberg, Victoria, Australia

^3^Department of Obstetrics, Centre for Perinatal Medicine, Division of Women and Child Health, University of Leipzig, Leipzig, Germany

^4^Department of Pharmacology and Therapeutics, University College Cork, Cork, Ireland

^5^Austin Health, Heidelberg, Victoria, Australia.

**Email addresses:**

Sabine Klein: sabine.klein@ukb.uni-bonn.de

Chandana Herath: [cherath@unimelb.edu.au](mailto:cherath@unimelb.edu.au)

Robert Schierwagen: [robert.schierwagen@ukb.uni-bonn.de](mailto:robert.schierwagen@ukb.uni-bonn.de)

Josephine Grace: [gracej@student.unimelb.edu.au](mailto:gracej@student.unimelb.edu.au)

Tom Haltenhof: [tom.haltenhof@uni-leipzig.de](mailto:tom.haltenhof@uni-leipzig.de)

Frank E. Uschner: [Frank.Uschner@ukb.uni-bonn.de](mailto:Frank.Uschner@ukb.uni-bonn.de)

Christian P. Strassburg: Christian.Strassburg@uni-bonn.de

Tilman Sauerbruch: [Tilman.Sauerbruch@ukb.uni-bonn.de](mailto:Tilman.Sauerbruch@ukb.uni-bonn.de)

Thomas Walther: [t.walther@ucc.ie](mailto:t.walther@ucc.ie)

Peter W. Angus: [Peter.ANGUS@austin.org.au](mailto:Peter.ANGUS@austin.org.au)

Jonel Trebicka: [jonel.trebicka@ukb.uni-bonn.de](mailto:jonel.trebicka@ukb.uni-bonn.de)

Corresponding author: Jonel Trebicka, Department of Internal Medicine I, University of Bonn, Sigmund-Freud Str. 25, D-53105 Bonn, Germany. [jonel.trebicka@ukb.uni-bonn.de](mailto:jonel.trebicka@ukb.uni-bonn.de), Tel: +49 228 287 15507, Fax: +49 228 287 19718

**Financial support:** The study was supported by grants from the Deutsche Forschungsgemeinschaft (SFB TRR57 P18; WA1441/22-2), J. & W. Hector- Foundation (M60.2) and National Health and Medical Research Council (NHMRC) of Australia (APP1008252).

**
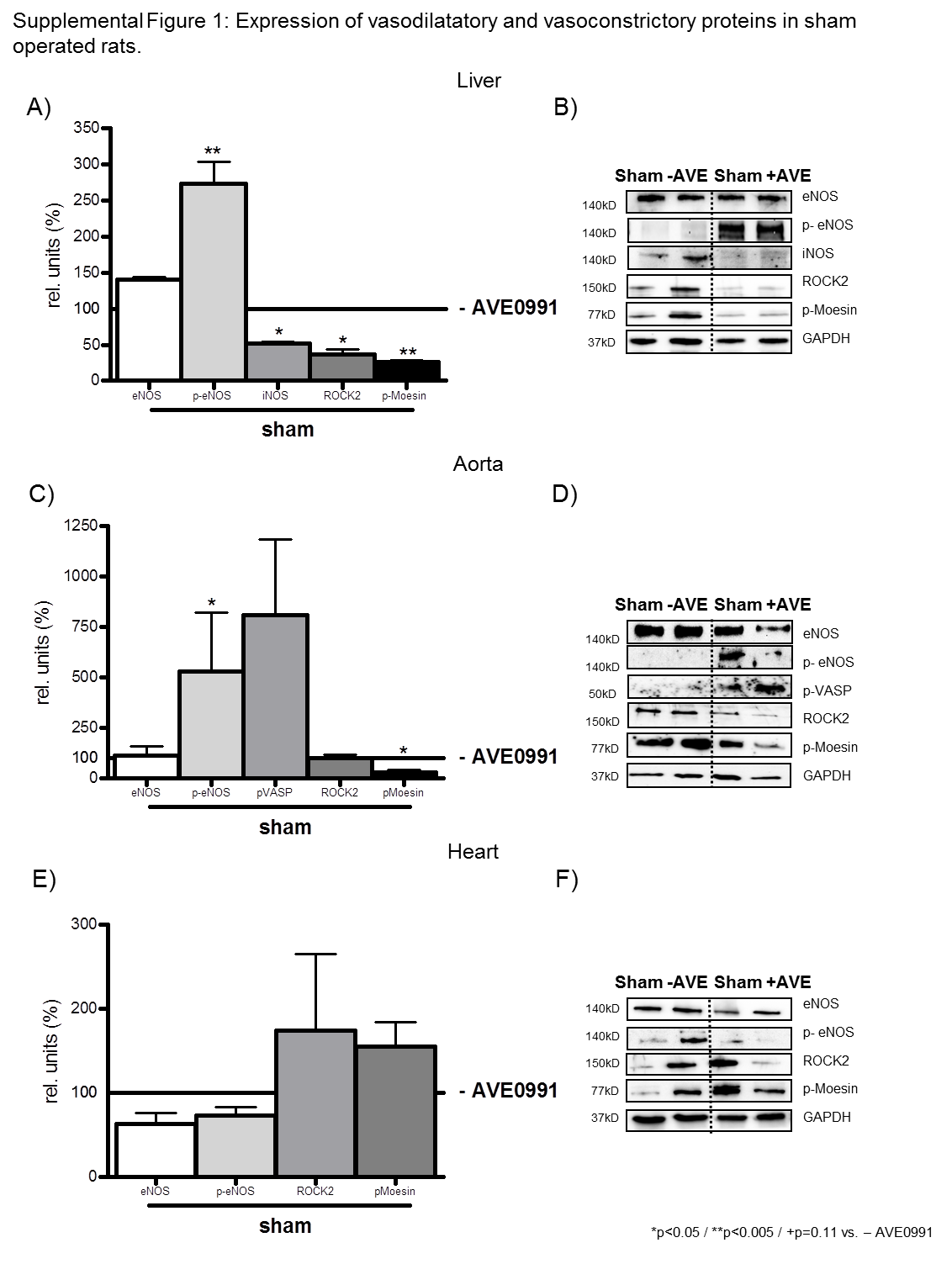
**

**Supplemental Figure A: Expression of vasodilatatory and vasoconstrictory proteins in sham operated rats.** (A, B) The hepatic protein expression of eNOS was not changed by AVE0991 injection in sham-operated rats. The protein expression of hepatic p-eNOS was significantly increased after AVE0991 injection in livers of control rats. AVE0991 injection decreased the hepatic protein levels of iNOS, ROCK2 and p-Moesin in sham operated rats. (C, D) In aortas of sham operated rats were no significant differences in protein expression levels of eNOS, pVASP and ROCK2. The protein expression of p-eNOS was increased in aortas of sham operated rats after AVE0991 injection. The activity of ROCK2, measured by the phosphorylation of it substrate Moesin was decreased after injection of AVE0991. (C, D). No significant differences in protein expression levels of eNOS, p-eNOS, ROCK2 and pMoesin were induced by AVE0991 injection in hearts of sham operated rats. All results are presented as quantifications and representive blots. Results were normalized to sham operated rats without AVE0991 injection.


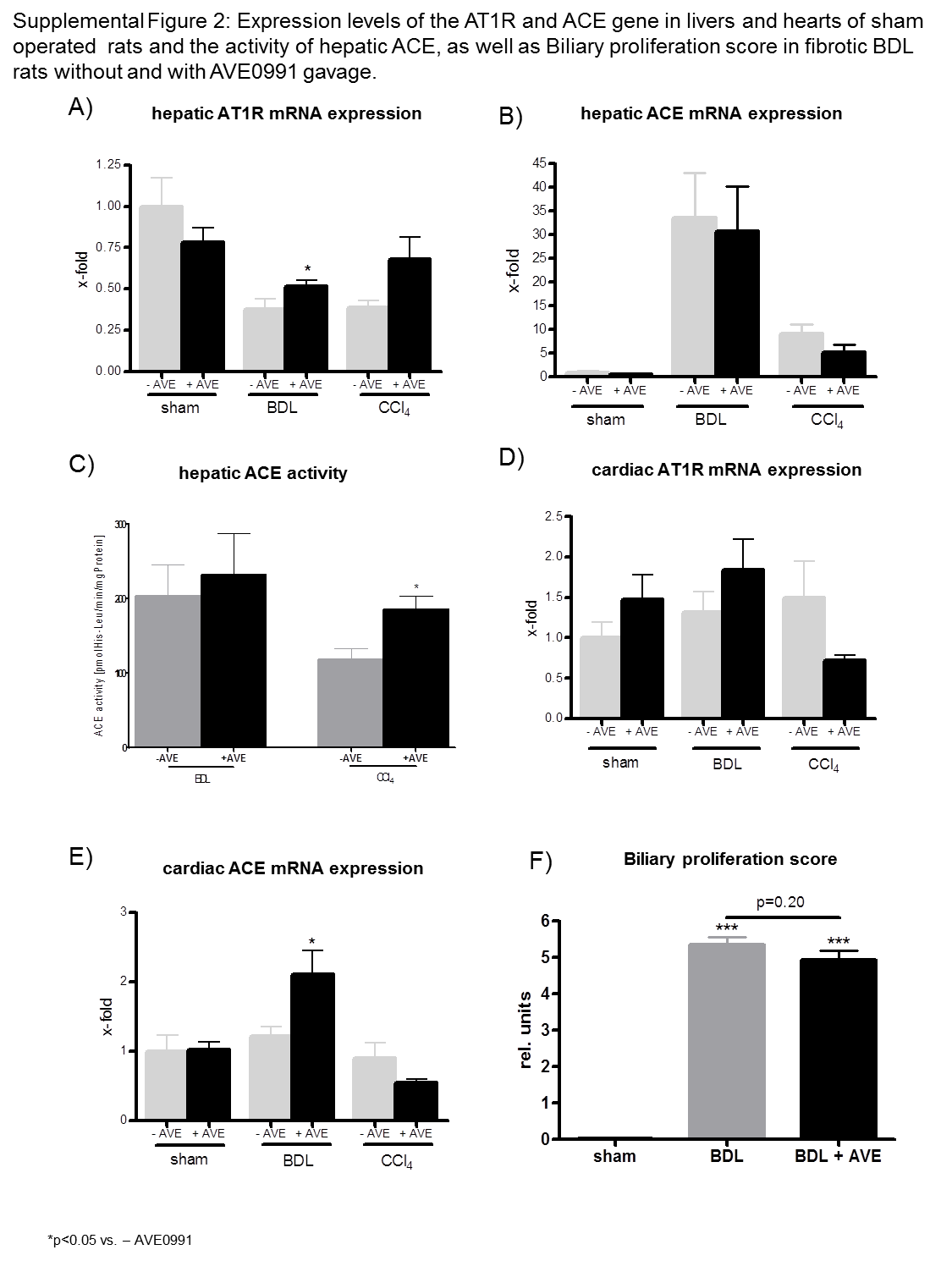


**Supplemental Figure B: Expression levels of AT1R and ACE mRNA expressions in livers and hearts of sham-operated rats and the activity of hepatic ACE and biliary proliferation score in fibrotic BDL rats without and with AVE0991 gavage.** (A) The hepatic mRNA expression of the AT1R was not influenced by injection of AVE0991 in sham-operated and in CCl_4_ intoxicated rats. In livers of BDL operated rats the AT1R mRNA was increased significantly after AVE0991 injection. (B) The hepatic mRNA expression of ACE was not changed by AVE0991 injection in sham-operated controls, nor in cirrhotic BDL or CCl_4_ intoxicated rats. (C) The activity of hepatic ACE was not modified by AVE0991 injection in cirrhotic BDL operated rats. The hepatic ACE activity was significantly increased by AVE0991 injection in CCl_4_ intoxicated rats. (D) The cardiac mRNA expression levels of the AT1R were not changed by AVE0991 injection in sham operated control rats and not in cirrhotic BDL or CCl_4_ intoxicated rats. (E) The cardiac mRNA expression of ACE was not influenced by AVE0991 injection in sham operated control rats nor in CCl_4_ intoxicated rats. In BDL rats, AVE0991 injection increased the cardiac ACE mRNA expression level. All results of mRNA expression levels are shown as the quantification of qRT-PCR results, normalized to sham-operated samples without AVE0991 injection. (F) The biliary proliferation score, used to describe the appearance of biliary epithelial cells in portal tracts, was increased in untreated BDL and in BDL rats after 2 weeks of AVE0991 treatment. There was no influence of AVE0991 treatment in the biliary proliferation score. Results are normalized to the biliary proliferation score in sham-operated rats.
