# Supplementary material for: Hemodynamic Effects of the Non-Peptidic Angiotensin-(1-7) Agonist AVE0991 in Liver Cirrhosis
Source: PLoS One. 2015 Sep 25;10(9):e0138732. doi: 10.1371/journal.pone.0138732 (PMC4583473; doi:10.1371/journal.pone.0138732)
Supplement: S1 Methods — (DOCX) [file pone.0138732.s002.docx]

Hemodynamic effects of the non-peptidic angiotensin-(1-7) agonist AVE0991 in liver cirrhosis

Sabine Klein¹*, Chandana B. Herath^2*^, Robert Schierwagen^1^, Josephine Grace^2^, Tom Haltenhof^3^, Frank E. Uschner^1^, Christian P. Strassburg¹, Tilman Sauerbruch^1^, Thomas Walther^3,4^, Peter W. Angus^5$^ and Jonel Trebicka¹^$+^

^*^ shared first authorship; ^$^ shared last authorship; ^+^ Corresponding author

**Affiliations:**

¹Department of Internal Medicine I, University of Bonn, Germany

^2^Department of Medicine, University of Melbourne, Austin Health, Heidelberg, Victoria, Australia

^3^Department of Obstetrics, Centre for Perinatal Medicine, Division of Women and Child Health, University of Leipzig, Leipzig, Germany

^4^Department of Pharmacology and Therapeutics, University College Cork, Cork, Ireland

^5^Austin Health, Heidelberg, Victoria, Australia.

**Email addresses:**

Sabine Klein: sabine.klein@ukb.uni-bonn.de

Chandana Herath: [cherath@unimelb.edu.au](mailto:cherath@unimelb.edu.au)

Robert Schierwagen: [robert.schierwagen@ukb.uni-bonn.de](mailto:robert.schierwagen@ukb.uni-bonn.de)

Josephine Grace: [gracej@student.unimelb.edu.au](mailto:gracej@student.unimelb.edu.au)

Tom Haltenhof: [tom.haltenhof@uni-leipzig.de](mailto:tom.haltenhof@uni-leipzig.de)

Frank E. Uschner: [Frank.Uschner@ukb.uni-bonn.de](mailto:Frank.Uschner@ukb.uni-bonn.de)

Christian P. Strassburg: Christian.Strassburg@uni-bonn.de

Tilman Sauerbruch: [Tilman.Sauerbruch@ukb.uni-bonn.de](mailto:Tilman.Sauerbruch@ukb.uni-bonn.de)

Thomas Walther: [t.walther@ucc.ie](mailto:t.walther@ucc.ie)

Peter W. Angus: [Peter.ANGUS@austin.org.au](mailto:Peter.ANGUS@austin.org.au)

Jonel Trebicka: [jonel.trebicka@ukb.uni-bonn.de](mailto:jonel.trebicka@ukb.uni-bonn.de)

Corresponding author: Jonel Trebicka, Department of Internal Medicine I, University of Bonn, Sigmund-Freud Str. 25, D-53105 Bonn, Germany. [jonel.trebicka@ukb.uni-bonn.de](mailto:jonel.trebicka@ukb.uni-bonn.de), Tel: +49 228 287 15507, Fax: +49 228 287 19718

**Financial support:** The study was supported by grants from the Deutsche Forschungsgemeinschaft (SFB TRR57 P18; WA1441/22-2), J. & W. Hector- Foundation (M60.2) and National Health and Medical Research Council (NHMRC) of Australia (APP1008252).

**Supplemental Methods.**

**Hepatic hydroxyproline content.** The hepatic collagen content was determined by measurement of 6-hydroxyproline content in hydrolysed liver samples [1]. Briefly, liver samples (approximately 200mg) were hydrolysed in 5mL of 6M HCl at 110^o^C for 24 hours. The hydrolysate was diluted with 5ml distilled water and filtered. A 200μL sample was then dissolved in isopropanol, incubated with chloramine T at room temperature for 4 minutes, followed by Ehrlich’s reagent in isopropyl alcohol at 60^0^C for 25 minutes. Hydroxyproline content was measured using a spectrophotometer at a wavelength of 570nm, with a standard curve generated using known concentrations of hydroxyproline. The results were corrected for liver density and expressed as μg/g liver.

**Hepatic ductal proliferation.** 0, <10% of portal areas involved; 1, 10%–50% of portal areas involved; 2, >50% of portal areas involved; 3, circumferential involvement of at least 50% of the portal area without significant expansion of portal tract; 4, circumferential involvement of at least 50% of the portal area with significant expansion of portal tract; 5, same as 4 plus bridging of the portal tracts in <20% of instances; and 6, same as 4 plus >20% of the portal tracts showing bridging involvement as described previously [2].

**qRT-PCR analysis.** All qPCR reactions were carried out by using multiplexing in which both the target gene and endogenous reference gene were amplified in a single well. The probes and primers were designed by using the Primer Express software program (PE Applied Biosystems, CA). Predeveloped TaqMan 18S ribosomal RNA kit was used as endogenous reference gene (PE Biosystems). Each sample was run and analyzed in duplicate. The normalized values from healthy liver tissues were used as the calibrator with a given value of 1, and the BDL groups were compared with this calibrator.

**Hemodynamic studies.** Briefly rats were anesthetized with ketamine/xylazine (78mg/kg and 10mg/kg body weight), respectively. This condition has been shown to approximate most closely the conscious state in terms of cardiac output and regional blood flow [3] and has been used extensively to investigate hemodynamic effects of drugs in animal models of portal hypertension [4–6]. The left femoral artery was cannulated for measurement of MAP and to obtain blood withdrawal. A cannula in the ileocecal vein pushed to the portal vein was used for portal pressure (PP) measurement. In the left femoral vein AVE0991 (1mg/kg) was injected. To inject microspheres, a cannula in the carotid and the cannula in the portal vein were taken.

**Microsphere technique.** Rats were fasted overnight but allowed free access to water. Median laparotomy was performed; a PE-50 catheter was introduced into a small ileocoecal vein and advanced to the portal vein for the measurement of PP. The left femoral artery was cannulated with PE-50 catheters for measurement of MAP and blood withdrawal. Via the right carotid artery, another PE-50 catheter was advanced into the left ventricle under pulse curve control. This catheter was used for microsphere application. The catheters in the femoral artery and the portal vein were connected to a pressure transducer (AD Instruments, Germany) for blood pressure measurement. The zero point was 1 cm above the operating table. After insertion of all catheters, rats were allowed to stabilize hemodynamically for 30min. A reference sample was obtained for 1min at a rate of 0.65mL/min before and after i.v. injection of AVE0991 (1mg/kg), using a continuous withdrawal pump (Hugo-Sachs-Elektronik,March-Hugstetten,Germany). Before injection of 1mg/kg AVE0991 in the femoral vein, 300,000 systemic (red) microspheres (15µm diameter, Triton-Technologies, San Diego, USA) were suspended in 0.3mL saline containing 0.05% Tween and injected in the left ventricle 10sec after the withdrawal pump had been started. Mesenteric portal-systemic shunt volume was estimated after injection of 150,000 white microspheres in 0.3ml saline containing 0.05% Tween in an ileocecal vein within 30 seconds [7]. 1h after injection of AVE0991 through the femoral vein 300,000 systemic (yellow) microspheres (15µm diameter, Triton-Technologies, San Diego, USA) were suspended in 0.3mL saline containing 0.05% Tween and injected in the left ventricle 10sec after the withdrawal pump had been started. Mesenteric portal-systemic shunt volume was estimated after injection of 150,000 blue microspheres in 0.3ml saline containing 0.05% Tween in an ileocecal vein within 30 seconds [7]. The blood reference probes were digested by addition of 3.8ml 5.3M KOH and 0.5ml Tween 80 and subsequent boiling for 1 hour. Tissues were cut in half and digested in 7ml 4M KOH with 20% Tween 80. The digested tissues and blood samples were vortexed and filtered using Whatman Nucleopore filters (Whatman International Ltd., Madison, UK). The colour of the filtered microspheres was dissolved in 0.2ml dimethyl-formamide, and the absorption was measured using spectrophotometry. Thereafter, cardiac output and organ blood flow before and 1h after AVE0991 injection were calculated using software obtained by Triton Technologies and expressed per 100g body weight. Splanchnic perfusion pressures were defined as MAP minus PP. Splanchnic-vascular resistances were calculated from the ratios between splanchnic perfusion pressures and the measured splanchnic blood flows, without including hepatic arterial flows. Mesenteric portal-systemic shuntflows were measured as the fraction of white/blue micropheres in the lung from total white/blue microspheres injected in an ileocolic vein. Hepatic portal-vascular resistances were estimated as PP divided by sum of gastrointestinal and splenic perfusions minus mesenteric portal-systemic shuntflow. Systemic-vascular resistances (SVR) were calculated as the ratio between MAP and cardiac output. Arterial-vascular resistances of specific organs (kidney, spleen, liver, stomach-gut) were calculated as the ratios between MAP and organ blood flows.

**Hepatic protein expression.** Primary antibodies were used in AVE0991 treated rat livers and their controls (Suppl. Table 2). Thereafter, the membranes were incubated with corresponding secondary peroxidase-coupled antibodies (Calbiochem, San Diego, USA). The housekeeping gene GADPH was used as loading control. Blots were developed with enhanced chemiluminescence (ECL, Amersham, UK). Intensities of the digitally detected bands were evaluated densitometrically using Chemi-Smart (PeqLab, Biotechnologies, Erlangen, Germany).

**ACE and ACE2 activity assay.** Using 10^-6^ M lisinopril the specifity was determined (Sigma Aldrich GmbH, Taufkirchen, Germany). The fluorescence from His-Leu after reaction with *o*-phthalaldehyde was detected at 360nm (excitation) and 485nm (emission). The activity is expressed as nmol His-Leu/min/mg protein.

As ACE2 substrate, Mca-APK (Dnp) (Biosnthan GmbH, Berlin, Germany) was dissolved in 100%DMSO to prepare a stock solution (10mM). Before assay the stock solution was diluted in 17% DMSO to the final concentration of 1mM. 15 µM ZnCl_2_ was given to each well and using the assay buffer (50mM 2-Morpholinoethanesulfonic acid, 300mM NaCl, 0.01% Brij-35, pH 6.5) the measurement was performed and started by adding 10µl tissue homogenate. The plate was tranfered to ice (4°C) and measured immediately. The reaction was suppressed after 1h of incubation (24 °C). Control tests were made in the presence of 1µM DX600 (Phoenix Europe GmbH, Karsruhe, Germany). Omni MMP^TM^ fluorogenic control peptide (Enzo Lifesciences, Lörrach, Germany) was used for standardization. Functionality was proven by a standardized solution with a defined, recombinant ACE2 activity (R&D Systems GmbH, Wiesbaden, Germany). Fluorescence was measured at 328/393nm.

**References:**

[1] Bergman I, Loxley R. The determination of hydroxyproline in urine hydrolysates. Clin Chim Acta 1970;27:347–9.

[2] Miyoshi H, Rust C, Roberts PJ, Burgart LJ, Gores GJ. Hepatocyte apoptosis after bile duct ligation in the mouse involves Fas. Gastroenterology 1999;117:669–77.

[3] Seyde WC, Longnecker DE. Anesthetic influences on regional hemodynamics in normal and hemorrhaged rats. Anesthesiology 1984;61:686–98.

[4] Trebicka J, Hennenberg M, Laleman W, Shelest N, Biecker E, Schepke M, et al. Atorvastatin lowers portal pressure in cirrhotic rats by inhibition of RhoA/Rho-kinase and activation of endothelial nitric oxide synthase. Hepatology 2007;46:242–53. doi:10.1002/hep.21673.

[5] Van de Casteele M, Sägesser H, Zimmermann H, Reichen J. Characterisation of portal hypertension models by microspheres in anaesthetised rats: a comparison of liver flow. Pharmacol Ther 2001;90:35–43.

[6] Trebicka J, Leifeld L, Hennenberg M, Biecker E, Eckhardt A, Fischer N, et al. Hemodynamic effects of urotensin II and its specific receptor antagonist palosuran in cirrhotic rats. Hepatology 2008;47:1264–76. doi:10.1002/hep.22170.

[7] Geraghty JG, Angerson WJ, Carter DC. Autoradiographic study of the regional distribution of gastric blood flow in portal hypertensive rats. Gastroenterology 1989;97:1108–14.
