# Supplementary material for: Hemodynamic Effects of the Non-Peptidic Angiotensin-(1-7) Agonist AVE0991 in Liver Cirrhosis
Source: PLoS One. 2015 Sep 25;10(9):e0138732. doi: 10.1371/journal.pone.0138732 (PMC4583473; doi:10.1371/journal.pone.0138732)
Supplement: S1 Tables — (DOCX) [file pone.0138732.s003.docx]

Hemodynamic effects of the non-peptidic angiotensin-(1-7) agonist AVE0991 in liver cirrhosis

Sabine Klein¹*, Chandana B. Herath^2*^, Robert Schierwagen^1^, Josephine Grace^2^, Tom Haltenhof^3^, Frank E. Uschner^1^, Christian P. Strassburg¹, Tilman Sauerbruch^1^, Thomas Walther^3,4^, Peter W. Angus^5$^ and Jonel Trebicka¹^$+^

^*^ shared first authorship; ^$^ shared last authorship; ^+^ Corresponding author

**Affiliations:**

¹Department of Internal Medicine I, University of Bonn, Germany

^2^Department of Medicine, University of Melbourne, Austin Health, Heidelberg, Victoria, Australia

^3^Department of Obstetrics, Centre for Perinatal Medicine, Division of Women and Child Health, University of Leipzig, Leipzig, Germany

^4^Department of Pharmacology and Therapeutics, University College Cork, Cork, Ireland

^5^Austin Health, Heidelberg, Victoria, Australia.

**Email addresses:**

Sabine Klein: sabine.klein@ukb.uni-bonn.de

Chandana Herath: [cherath@unimelb.edu.au](mailto:cherath@unimelb.edu.au)

Robert Schierwagen: [robert.schierwagen@ukb.uni-bonn.de](mailto:robert.schierwagen@ukb.uni-bonn.de)

Josephine Grace: [gracej@student.unimelb.edu.au](mailto:gracej@student.unimelb.edu.au)

Tom Haltenhof: [tom.haltenhof@uni-leipzig.de](mailto:tom.haltenhof@uni-leipzig.de)

Frank E. Uschner: [Frank.Uschner@ukb.uni-bonn.de](mailto:Frank.Uschner@ukb.uni-bonn.de)

Christian P. Strassburg: Christian.Strassburg@uni-bonn.de

Tilman Sauerbruch: [Tilman.Sauerbruch@ukb.uni-bonn.de](mailto:Tilman.Sauerbruch@ukb.uni-bonn.de)

Thomas Walther: [t.walther@ucc.ie](mailto:t.walther@ucc.ie)

Peter W. Angus: [Peter.ANGUS@austin.org.au](mailto:Peter.ANGUS@austin.org.au)

Jonel Trebicka: [jonel.trebicka@ukb.uni-bonn.de](mailto:jonel.trebicka@ukb.uni-bonn.de)

Corresponding author: Jonel Trebicka, Department of Internal Medicine I, University of Bonn, Sigmund-Freud Str. 25, D-53105 Bonn, Germany. [jonel.trebicka@ukb.uni-bonn.de](mailto:jonel.trebicka@ukb.uni-bonn.de), Tel: +49 228 287 15507, Fax: +49 228 287 19718

**Financial support:** The study was supported by grants from the Deutsche Forschungsgemeinschaft (SFB TRR57 P18; WA1441/22-2), J. & W. Hector- Foundation (M60.2) and National Health and Medical Research Council (NHMRC) of Australia (APP1008252).

**Supplemental Table A: Primer and probes used for qRT-PCR.** ACE, ACE2, AT1R, MasR, αSMA, Col I and CTGF were detected by qRT-PCR with the listed probes and primers.

**Supplemental Table B: Primary antibodies used for western blots.** List of the primary antibodies to detect the protein expression levels by western blots.

**Supplemental Table C:** **Hemodynamic parameters before and after AVE0991 injection.** Table of hemodynamic parameters before and after AVE0991 injection in control, BDL and CCl_4_ rats. The splanchnic-vascular resistance was increased after AVE0991 injection in CCl_4_ intoxicated rats. The systemic-vascular resistance was not altered in control, BDL and CCl_4_ rats by injection of AVE0991. The cardiac output was increased after liver cirrhosis induction by BDL and CCl_4_, but not influenced by AVE0991 injection.
